# The depression GWAS risk allele predicts smaller cerebellar gray matter volume and reduced *SIRT1* mRNA expression in Chinese population

**DOI:** 10.1038/s41398-019-0675-3

**Published:** 2019-12-09

**Authors:** Weipeng Liu, Hao Yan, Danyang Zhou, Xin Cai, Yuyanan Zhang, Shiyi Li, Huijuan Li, Shiwu Li, Dong-Sheng Zhou, Xingxing Li, Chen Zhang, Yan Sun, Jia-Pei Dai, Jingmei Zhong, Yong-Gang Yao, Xiong-Jian Luo, Yiru Fang, Dai Zhang, Yina Ma, Weihua Yue, Ming Li, Xiao Xiao

**Affiliations:** 1 Key Laboratory of Animal Models and Human Disease Mechanisms of the Chinese Academy of Sciences and Yunnan Province, Kunming Institute of Zoology, Chinese Academy of Sciences, Kunming, Yunnan China; 2 Kunming College of Life Science, University of Chinese Academy of Sciences, Kunming, Yunnan China; 3 Peking University Sixth Hospital/Institute of Mental Health, Beijing, China; 4 NHC Key Laboratory of Mental Health (Peking University) and National Clinical Research Center for Mental Disorders (Peking University Sixth Hospital), Beijing, China; 5 State Key Laboratory of Cognitive Neuroscience and Learning, IDG/McGovern Institute for Brain Research, Beijing Normal University, Beijing, China; 6 Department of Psychiatry, Ningbo Kangning Hospital, Ningbo, Zhejiang China; 7 Shanghai Mental Health Center, Shanghai Jiao Tong University School of Medicine, Shanghai, China; 8 Wuhan Institute for Neuroscience and Neuroengineering, South-Central University for Nationalities, Wuhan, Hubei China; 9 Chinese Brain Bank Center, Wuhan, Hubei China; 10 Psychiatry Department, The first people’s hospital of Yunnan province, Kunming, Yunnan China; 11 CAS Center for Excellence in Brain Science and Intelligence Technology, Chinese Academy of Sciences, Shanghai, China; 12 KIZ/CUHK Joint Laboratory of Bioresources and Molecular Research in Common Diseases, Kunming, Yunnan China; 13 Center for Excellence in Animal Evolution and Genetics, Chinese Academy of Sciences, Kunming, Yunnan China; 14 Peking-Tsinghua Joint Center for Life Sciences and PKU IDG/McGovern Institute for Brain Research, Peking University, Beijing, China

**Keywords:** Genomics, Depression

## Abstract

Major depressive disorder (MDD) is recognized as a primary cause of disability worldwide, and effective management of this illness has been a great challenge. While genetic component is supposed to play pivotal roles in MDD pathogenesis, the genetic and phenotypic heterogeneity of the illness has hampered the discovery of its genetic determinants. In this study, in an independent *Han* Chinese sample (1824 MDD cases and 3031 controls), we conducted replication analyses of two genetic loci highlighted in a previous Chinese MDD genome-wide association study (GWAS), and confirmed the significant association of a single nucleotide polymorphism (SNP) rs12415800 near *SIRT1*. Subsequently, using hypothesis-free whole-brain analysis in two independent *Han* Chinese imaging samples, we found that individuals carrying the MDD risk allele of rs12415800 exhibited aberrant gray matter volume in the left posterior cerebellar lobe compared with those carrying the non-risk allele. Besides, in independent *Han* Chinese postmortem brain and peripheral blood samples, the MDD risk allele of rs12415800 predicted lower *SIRT1* mRNA levels, which was consistent with the reduced expression of this gene in MDD patients compared with healthy subjects. These results provide further evidence for the involvement of *SIRT1* in MDD, and suggest that this gene might participate in the illness via affecting the development of cerebellum, a brain region that is potentially underestimated in previous MDD studies.

## Introduction

Major depressive disorder (MDD), a clinically and genetically heterogeneous illness, has led to significant social and economic burden worldwide, and tremendous efforts have been invested to investigate its underlying pathological mechanisms in the past decades^1^. Convergent findings have pointed to the involvement of dendritic spine pathology, synaptic dysfunction, as well as aberrant structure and function of prefrontal cortex and hippocampus in the neurobiology of MDD^2–6^. Besides, accumulating data also highlights additional brain regions in its pathogenesis, such as cerebellum, which is engaged in emotional and cognitive processes^7,8^. In addition to these basic and preclinical findings, scientists have also obtained strong evidence supporting the unneglectable role of genetic susceptibility factors in the pathogenesis of MDD, whose heritability has been estimated to be ~37%^9^, and multiple genomic loci were found to be significantly associated with the illness in populations of European origin^10,11^. For example, a recent meta-analysis of genome-wide association study (GWAS) datasets in Europeans reported that 102 independent loci spanning 269 genes were significantly associated with depression, and many of them were linked to synaptic structure and neurotransmission^11^. Besides, studies dissecting the genetic architectures of MDD in other populations, e.g., *Han* Chinese, are also emerging in recent years. For instance, a *Han* Chinese sparse whole-genome sequencing study of 10,640 female subjects followed by independent replications in 6417 individuals of both sexes identified two single nucleotide polymorphisms (SNPs) conferring risk of MDD. One SNP (rs12415800) was near the *Sirtuin 1* gene (*SIRT1*), and the other one (rs35936514) was in an intron of *LHPP* (this study was named CONVERGE GWAS)^12^. However, neither SNP showed evidence of association with risk of MDD in populations of European ancestry (rs12415800, *p* = 0.797; rs35936514, *p* = 0.293)^10^, and rs12415800 was even near monomorphic in Europeans (frequency of A-allele, 0.023 in Europeans versus 0.401 in Chinese, according to genotype data from 1000 Genomes Project^13^). Thus, this CONVERGE GWAS is believed to provide essential knowledge primarily regarding the genetic components of MDD in *Han* Chinese^12^.

However, statistical associations between genetic markers and clinical diagnosis in GWAS do not directly reveal their underlying mechanisms^14–16^, it is thus essential to translate genetic risk into neural mechanisms using biological approaches. Gene editing in murine models, which provides important clues for the function of MDD-risk genes (such as *SIRT1*)^17,18^, may be insufficient to fully characterize the disease mechanisms as human brains are more complicated than murine brains, and in vivo magnetic resonance imaging (MRI) analyses in living humans are believed to provide essential information. Recent MRI studies showed that MDD patients displayed abnormalities in subcortical brain structures compared with healthy controls^19,20^, and the relatives of MDD patients (individuals at increased genetic risk) exhibited similar deficits in phenotypes with less severity^21,22^. Therefore, these phenotypes likely reflect the biological pathways directly linked to the genetic risk factors of MDD^14–16^. A plausible strategy, to translate statistical associations between genetic loci and clinical diagnosis of MDD into potential neural mechanisms, is thus proposed to identify effects of genetic risk loci in the brain in virtue of such endophenotypic analyses^23–30^. To date, effects of the CONVERGE MDD GWAS loci on such phenotypes have been scarcely reported in *Han* Chinese, excepting a study showing associations between *SIRT1* SNPs and regional cortical gray matter density in 92 healthy individuals from Eastern China^31^. Hence, to characterize the neural mechanisms underlying putative genetic risk loci in the CONVERGE MDD GWAS^12^, the first aim of the present study is to examine their effects on regional gray matter volumes (GMV) in *Han* Chinese individuals using structural MRI approaches.

Meanwhile, the biological impacts of most GWAS risk loci remain unclear as they mainly reside in the noncoding regions of the genome. Accumulating evidence suggests that these noncoding loci tend to affect mRNA expression of particular genes^32^. Indeed, altered expression of certain genes have been reported in the brain or peripheral blood of MDD patients compared with healthy controls^33–35^. For example, *SIRT1* mRNA levels were previously found significantly reduced in the peripheral blood of MDD patients^36–38^. Nevertheless, whether such genes are relevant to the genetic susceptibility of MDD is unclear. Therefore, the second aim of the present study is to test whether the MDD risk SNP rs12415800 was associated with altered mRNA expression levels of certain genes in human brain and peripheral blood tissues.

## Methods and materials

All the protocols and methods were approved by the institutional review board of Kunming Institute of Zoology, Chinese Academy of Sciences, and the ethics committees of all participating hospitals and universities. Informed consents were obtained from all participants prior to the study.

### MDD case-control sample and statistical analysis in Chinese population

1824 MDD cases and 3031 controls of Chinese origin were recruited from Mainland China. Briefly, each MDD patient was diagnosed in mental health centers strictly following the DSM-IV guidelines in combination with clinical information collected through medical record review and family member interviews. Subjects affected by other psychiatric disorders or neurological disorders, being or planning to be pregnant, or breast-feeding at the time of study, were excluded. Control subjects were local volunteers with no self-reported history of mental illnesses. The samples we collected were not overlapped with the samples used in previous MDD CONVERGE GWAS^12^.

DNA samples were randomly distributed in plates and genotyped using the SNaPShot method, and all assays were performed blind to diagnosis and genotype. Logistic regression was utilized to analyze the associations between SNPs and MDD, with sex and residence of participants included in the covariates. For meta-analysis, we retrieved odds ratio (OR) and standard error (SE) values of each individual sample to calculate the inter-sample heterogeneity, pooled OR, and the overall 95% confidence intervals (CIs). We considered SNPs with a one-tailed *p* < 0.05 in our primary sample to be nominally significant; in the overall meta-analysis, SNPs with a two-tailed *p* < 5.00 × 10^−8^ was considered genome-wide statistically significant. Power analysis was conducted using the Power and Sample Size Program software^39^, and the reported OR of 1.150 in CONVERGE MDD GWAS^12^ was applied in the power analysis, which corresponds to a “weak” gene effect.

### Structural imaging analysis in Chinese population

We used two independent structural imaging samples of *Han* Chinese population, the discovery sample (Beijing sample) and replication sample (Kunming sample).

#### Discovery sample

508 unrelated healthy controls (258 females, 250 males, mean age 24.5 ± 4.0 years) were recruited from the local community and screened using the Structured Clinical Interview for Diagnostic and the Statistical Manual of Mental Disorders, Fourth Edition, Text Revision (DSM-IV-TR) Axis I Disorders (SCID, non-patient edition). All participants were right-handed *Han* Chinese without any lifetime history or family history of psychiatric disorders. Magnetic resonance (MR) images were acquired using a 3.0T GE Discovery MR750 scanner at the Center for MRI Research, Peking University. T1-weighted high-resolution structural image was acquired in a sagittal orientation using an axial 3D fast, spoiled gradient recalled (FSPGR) sequence with the following parameters: repetition time = 6.66 ms, echo time = 2.93 ms, field of view = 256 × 256 mm^2^, slice thickness/gap = 1.0/0 mm, acquisition voxel size = 1 × 1 × 1 mm^3^, flip angle = 12°, 192 contiguous sagittal slices.

#### Replication sample

262 unrelated healthy *Han* Chinese subjects (174 females, 88 males, mean age 33.8 ± 12.0 years) were recruited for the current study. Healthy controls were recruited and interviewed to ensure that no one had lifetime history of psychiatric disorders, or received any treatment for psychiatric disorders. Structural MRI data were acquired using a Philips MRI scanner (Achieva Release 3.2.1.0) operating at 3 T, and high-resolution whole-brain T1-weighted images were acquired sagittally with an inversion-recovery prepared 3-D spoiled gradient echo (SPGR) pulse sequence (repetition time = 7.38 ms, echo time = 3.42 ms, flip angle = 8°, voxel dimensions = 1.04 ⨯ 1.04 ⨯ 1.80 mm^3^, slice thickness = 1.2 mm).

#### Statistical analysis

In both MRI samples, the structural images were processed with DPABI (http://rfmri.org/DPABI), a MatLab toolbox that calls for statistical parametric mapping 8 (SPM8, http://www.fil.ion.ucl.ac.uk/spm). Diffeomorphic Anatomical Registration Through Exponentiated Lie algebra (DARTEL) toolbox was also used to perform voxel-based morphometry (VBM) analysis with default parameters. All images were then normalized to the standard Montreal Neurological Institute (MNI) template, modulated to account for volume changes in the warping, and resampled to 1.5 ⨯ 1.5 ⨯ 1.5 mm^3^. Modulated gray matter images were smoothed with an 8 mm Gaussian kernel. An explicit mask was used from the SPM intracranial brain template so as to restrict which voxels should undergo statistical analysis. Results of different genotypic groups were compared using one-way ANOVA model with sex, age, and total GMV as covariates. We considered a whole-brain family-wise error (FWE) correction *p* < 0.05 with a cluster size>10 as an authentic significant effect.

### Expression quantitative trait loci (eQTL) analysis of SIRT1 mRNA expression in Chinese brain samples

#### Discovery sample

Frozen amygdala tissues of 65 non-psychiatric individuals were obtained from the Chinese Brain Bank Center^40,41^. The RNA and DNA extractions, cDNA synthesis and quantitative real-time PCR (qRT-PCR) were performed as previously described^40^. In brief, total RNA was isolated from the amygdala tissues using TRIzol reagent (Life Technologies, USA). Gene expression levels were quantified using qRT-PCR with SYBR green mix (Roche, USA). The primer sequences used for human *SIRT1* amplification were 5′-TCGCAACTATACCCAGAACATAGACA-3′ (forward) and 5′-CTGTTGCAAAGGAACCATGACA-3′ (reverse), and sequences of primers for the housekeeping gene *RPS13* were 5′-CCCCACTTGGTTGAAGTTGA-3′ (forward) and 5′-CTTGTGCAACACCATGTGAA-3′ (reverse). The qRT-PCR assays were performed in triplicates, results were normalized to the expression of *RPS13* and mean 2^–ΔΔCt^ values (relative to one genotypic group) were calculated for each subject as the relative gene expression levels. Statistical test against genotypic groups was performed using one-way ANCOVA analysis, adjusting for age, gender and RNA integrity number (RIN).

#### Replication sample

Frozen amygdala tissues from 72 non-psychiatric donors were collected as the replication samples under the same criteria as those for the discovery sample. RNA isolation and gene expression quantification were then performed as described above.

### Diagnostic analysis of SIRT1 mRNA expression in Chinese peripheral blood samples

Fifty unrelated first-episode drug-naive MDD patients (all were diagnosed following the DSM-V guidelines, and were not taking medications) and 52 healthy control subjects were recruited from the First people’s hospital of Yunnan province. MDD cases with substance abuse or other co-occurring mental disorders were excluded, and the 17-item Hamilton Rating Scale for Depression (HAMD17) was used to evaluate the depression level. Controls were local volunteers without physical or mental illnesses. Details of the sample information have been described in a recent study^42^. RNA extraction, cDNA synthesis and qRT-PCR were performed as described above. The relative gene expression was presented as the means of 2^–ΔΔCt^ (relative to the control sample or one genotypic group), and one-way ANCOVA analysis was used to test if *SIRT1* was significantly altered in MDD cases compared with controls, as well as between different genotypic groups.

## Results

### Rs12415800 is significantly associated with MDD

The previous *Han* Chinese MDD GWAS of 10,640 female individuals identified several SNPs showing genome-wide associations with MDD, and two of them (rs12415800 and rs35936514) were also significantly associated with MDD in an independent sample of both sexes (6417 subjects)^12^. In an attempt to further replicate the associations of rs12415800 and rs35936514 with the risk of MDD, we independently recruited 1824 MDD cases and 3031 controls from mainland China. There is no overlap between our primary MDD case-control sample and the samples utilized in previous CONVERGE GWAS^12^. Both SNPs were in Hardy–Weinberg Equilibrium in cases and controls (*p* > 0.05). Notably, the putative MDD risk allele (A) of rs12415800 showed a marginally significant overrepresentation in cases compared with controls (one-tailed *p* = 0.031, OR = 1.085, Table 1). This association signal and the direction of allelic effects were consistent with the previous GWAS^12^. We also conducted a power analysis of our primary MDD sample size using the following assumptions: 1824 MDD patients and 3031 controls, two-tailed *p* = 0.05, the frequency of rs12415800 A-allele in Chinese populations according to 1000 Genomes Project (0.401)^13^, and the reported OR of rs12415800 in CONVERGE GWAS (1.150)^12^. Our primary MDD sample size revealed a 64.3% power of detecting a significant association. Given that this primary MDD sample had a relatively lower statistical power, we performed a meta-analysis using data obtained from all available *Han* Chinese samples (i.e., discovery and replication samples in CONVERGE GWAS^12^), and observed a stronger association between rs12415800 and MDD (two-tailed *p* = 7.03 ⨯ 10^−11^, OR = 1.137, Table 2). However, rs35936514 was not associated with MDD in our primary sample (one-tailed *p* = 0.500), and was thus excluded from subsequent analyses. The genotype frequencies of the two SNPs are shown in Table S1.

**Table 1:** Association of CONVERGE GWAS SNPs with MDD in our primary Chinese sample (1824 cases and 3301 controls).

CHR	SNP	Position	Allele	Frequency		Two-tailed *p*-value	One-tailed *p*-value	OR	95%CIs
				Case	Control				
10	rs12415800	69624180	A/G	0.453	0.436	0.062	0.031	1.085	0.996-1.183
10	rs35936514	126244970	T/C	0.261	0.263	0.999	0.500	1.000	0.909-1.100

*CHR* chromosome, *SNP* single nucleotide polymorphism, *Allele* effect allele/non-effect allele, *Frequency* frequency of effect allele, *OR* odds ratio, *CIs* confidence intervals

Test of Hardy–Weinberg Equilibrium for rs12415800: case, *p* = 0.836; control, *p* = 0.408

Test of Hardy–Weinberg Equilibrium for rs35936514: case, *p* = 0.066; control, *p* = 0.612

**Table 2:** Meta-analysis of rs12415800 A-allele with MDD in *Han* Chinese population.

Sample	Case	Control	*p*-value	OR	95%CIs
CONVERGE Discovery	5303	5337	1.92 ⨯ 10^−8^	1.164	1.102–1.230
CONVERGE Replication	3231	3186	7.71 ⨯ 10^−4^	1.130	1.053–1.213
Current study	1824	3301	0.062	1.085	0.996–1.183
Meta-analysis	10,358	11,824	7.03 ⨯ 10^−11^	1.137	1.094–1.182

*OR* odds ratio, *CIs* confidence intervals

Test of heterogeneity for meta-analysis: *I*^*2*^ = 0, *p*-value = 0.390

### Rs12415800 is significantly associated with cerebellar gray matter volume

While the statistical association provided strong evidence for a putative role of rs12415800 in the pathogenesis of MDD, we further delved into potential underlying neural mechanisms. It was proposed that aberrant brain development might cause deficits in specific brain regions, leading to the onset of psychiatric illnesses including MDD^3^. We therefore examined whether rs12415800 was linked to alterations in the brain structure detected by in vivo MRI in two independent *Han* Chinese samples.

**Fig. 1:**
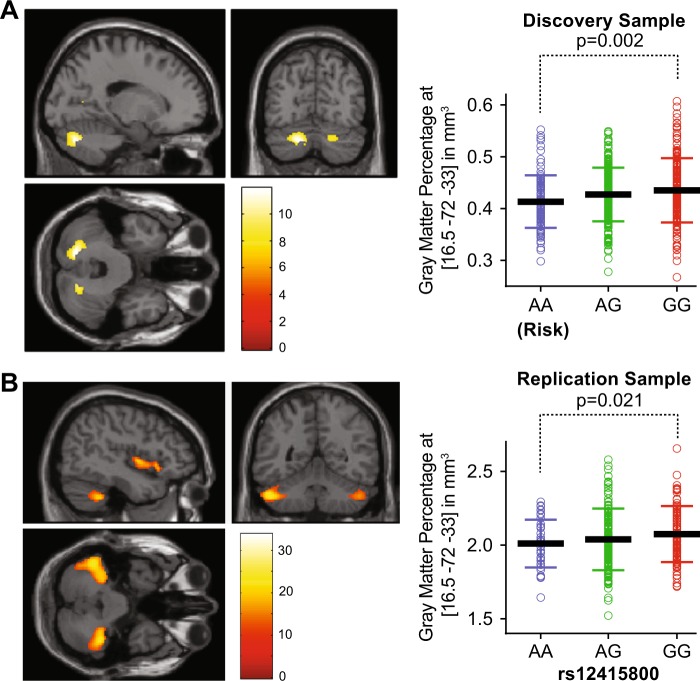
The MDD risk allele of rs12415800 showed significantly reduced gray matter volume (GMV) in the left posterior cerebellar lobe during whole-brain analysis in the Beijing (a) and Kunming (b) imaging samples. The *p*-values in the right dot plot were LSD correction *p*-values.

In our discovery imaging sample of 508 healthy subjects (acquired in Beijing), the whole-brain VBM analysis revealed significantly reduced GMV of the left posterior cerebellar lobe in the subjects carrying the MDD risk A-allele at both chromosomes compared with the other genotypic groups (peak voxel −16.5/−72/−33, *F* = 11.855, cluster size = 795, FWE corrected *p* = 0.015, Fig. 1a). Post-hoc analysis (removing subjects with extreme value which is 0.6115 that beyond mean ± 3*SD) with LSD correction indicated that the GMV in the left posterior cerebellar lobe was smaller in A/A than that in G/G genotype carriers (corrected *p* = 0.002, Fig. 1a) and that in A/G genotype carriers (corrected *p* = 0.048, Fig. 1a).

In an independent replication imaging sample including 262 healthy individuals (acquired in Kunming), the whole-brain VBM analysis also revealed significantly reduced GMV in the left posterior cerebellar lobe in the A/A carriers (MDD risk) compared with the other genotypic groups (peak voxel −49.5/−51/−40.5, *F* = 24.021, cluster size = 1647, FWE corrected *p* < 0.01, Fig. 1b), although the precise peak coordinates were not the same between the two samples. In the replication sample, we further analyzed the region of interest (ROI) from a sphere with a 10 mm radius centered at the peak voxel in discovery Beijing sample (−16.5/−72/−33) so as to directly replicate the results obtained from the discovery sample. The regional GMV in the left posterior cerebellar ROI (−16.5/−72/−33) was submitted to ANCOVA with genotype as between-subjects factor, and age, gender and the total GMV as covariates. Intriguingly, we again observed a significant inter-group difference in the omnibus test (*F*(2,255) = 3.503, corrected *p* = 0.032, Fig. 1b). Post-hoc analysis with LSD correction indicated that the GMV in the left posterior cerebellar lobe of G/G carriers was larger than that of the A/A (corrected *p* = 0.021, Fig. 1b) and A/G genotype subjects (corrected *p* = 0.031, Fig. 1b).

We also examined the effect of rs12415800 on GMV using data from imaging consortia such as ENIGMA and UK Biobank^43,44^. However, rs12415800 is almost monomorphic in European populations, and the data from these large consortia, which primarily analyzed European individuals, did not provide valuable information. Although the role of the left posterior cerebellar lobe region is yet to be characterized in mental illnesses, growing evidence has implied the involvement of cerebellar dysfunction in MDD^45^ in addition to its primary roles in motor control. Changes in the GMV of cerebellum have been identified in MDD^46^, and significant associations between cerebellar morphology and volume and cognitive performance were also reported^8,47^.

### Rs12415800 is associated with brain SIRT1 mRNA expression

**Fig. 2:**
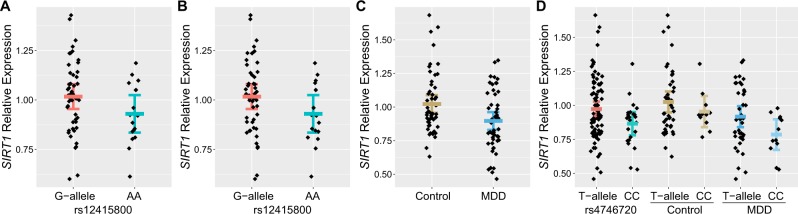
Effects of rs12415800 and MDD diagnostic status on *SIRT1* mRNA expression. **a** Association of rs12415800 with *SIRT1* mRNA expression in the discovery *Han* Chinese amygdala sample (*N* = 65). The values on *Y*-axis were presented as 2^–ΔΔCt^. **b** Association of rs12415800 with *SIRT1* mRNA expression in the replication *Han* Chinese amygdala sample (*N* = 72). The values on *Y*-axis were presented as 2^–ΔΔCt^. **c** Diagnostic analysis of *SIRT1* mRNA expression in the peripheral blood of 50 first-episode drug-naive MDD patients versus 52 healthy controls from *Han* Chinese. The values on *Y*-axis were presented as 2^–ΔΔCt^. **d** Association of rs4746720 (in high LD with rs12415800) with *SIRT1* mRNA expression in the blood samples. The values on *Y*-axis were presented as 2^–ΔΔCt^. Error bar represents ± SEM.

GWAS loci of complex diseases often exert their functions through affecting gene expression^32,48,49^. To understand whether rs12415800 was related to the expression of nearby genes, we conducted an eQTL analysis between the SNP and *SIRT1* expression using qRT-PCR methods in two independent samples of *Han* Chinese amygdala tissues, a brain region engages in emotion processing and has been frequently found abnormal in MDD patients^20,50^. In our discovery amygdala sample (*N* = 65), although the risk allele [A] carriers tended to show decreased *SIRT1* expression, the correlation was not statistically significant likely due to the small sample size in each genotypic group (one-tailed *p* = 0.0849). We then compared the mRNA expression of *SIRT1* between the risk allele homozygous group [A/A] and the other genotypic groups [A/G + G/G], we found that the expression of *SIRT1* was significantly lower in A/A group (MDD risk allele homozygotes) than A/G+G/G group (one-tailed *p* = 0.0391, Fig. 2a). In our replication amygdala sample (*N* = 72), the A/A genotype again indicated a lower expression of *SIRT1* (one-tailed *p* = 0.0963, Fig. 2b). Despite the relatively small size of each Chinese amygdala sample, the consistent direction of allelic effects across samples suggested a tight link between rs12415800 and *SIRT1* mRNA expression. To maximize the statistical power, we conducted a meta-analysis by combining the discovery and replication samples and observed a stronger association (one-tailed *p* = 0.0149). We have also queried the SNP rs12415800 in the public brain eQTL datasets, such as BrainSeq^51^, Brain xQTL^52^, CommonMind^53^, and PsychENCODE^54^, which primarily included individuals of European and African American ancestries. Unfortunately, rs12415800 or its linkage disequilibrium (LD) SNPs were not covered in these datasets likely due to the divergent allelic frequencies of this SNP in different populations (frequency of A-allele, 0.023 in Europeans versus 0.401 in Chinese, according to genotype data from 1000 Genomes Project^13^). Therefore, the eQTL associations of rs12415800 might be *Han* Chinese specific, and further analyses of this SNP in large Han Chinese cohorts are needed.

### Expression of SIRT1 is significantly reduced in MDD patients compared with healthy controls

Previous studies have reported lower *SIRT1* mRNA expression in the peripheral blood of MDD patients than in that of healthy controls in Chinese and European populations^36–38^. To validate this result, we collected peripheral blood tissues from an independent *Han* Chinese cohort (50 MDD cases and 52 controls) and tested their *SIRT1* mRNA expression using qRT-PCR. Notably, the cases in this sample were first-episode MDD patients who had not received any medication by the time of the blood collection. We found that *SIRT1* mRNA expression was decreased by 12.3% in the peripheral blood of MDD patients compared with controls (one-tailed *p* = 0.00284, Fig. 2c), which was consistent with previous results^36–38^. Therefore, reduced mRNA expression of *SIRT1* is likely a risk factor for MDD.

We then stratified the blood samples according to the risk genotypes. Since the genotyping results of rs12415800 are not directly available in this sample, we examined a SNP (rs4746720) in strong LD with rs12415800 in *Han* Chinese (*r*^2^ = 0.961, *D*′ = 1.000, according to genotype data from 1000 Genomes Project^13^). Interestingly, rs4746720 was also associated with *SIRT1* expression in the 102 *Han* Chinese blood tissues, with the C/C genotype carriers (which is linked with the risk allele homozygous group [A/A] at rs12415800) showing lower *SIRT1* mRNA levels compared with other groups (one-tailed *p* = 0.0213, Fig. 2d). In a further analysis of solely MDD cases or solely the controls, the C/C genotype of rs4746720 also predicted reduced *SIRT1* expression (one-tailed *p* = 0.0334 in MDD cases and one-tailed *p* = 0.181 in healthy controls, Fig. 2d). Notably, the above analyses results did not achieve the conventional significance level, likely due to the small sample size.

## Discussion

Due to the moderate heritability, great etiological and phenotypic heterogeneity, and limited knowledge of genotype–phenotype relationships, the genetic foundation of MDD remains difficult to elucidate in the past few years. Despite multiple GWASs in European populations, GWAS of MDD in *Han* Chinese populations has only been conducted once by the CONVERGE consortium^12^, in which they identified two GWAS loci of interest. The gene near one of these risk loci, *SIRT1*, has 9 exons and spans 33,715 bp at the chromosome 10q21.3 region. The association between MDD and this gene was successfully replicated in our independent *Han* Chinese samples and remained genome-wide statistically significant in the overall meta-analysis. This gene was also associated with MDD in Japanese^55^, although the risk SNPs were different between Japanese and *Han* Chinese. However, *SIRT1* was not highlighted as a MDD risk gene in European populations. This inconsistency between populations might be explained by several possibilities. A most likely explanation is that there might be fundamental differences of the genetic architecture at this locus between populations. Specifically, the allele frequencies at rs12415800 between *Han* Chinese and Europeans are largely divergent, and the LD structures linked with rs12415800 were also sharply distinct between the two populations (we observed a series of SNPs in strong LD with rs12415800 among *Han* Chinese, few of which were highly linked in Europeans (Fig. S1)). These distinctions could be attributed to the natural selection and different population histories, and their contributions to the inconsistent genetic risk factors for psychiatric illnesses between continental populations are widely supported by previous studies^56,57^.

*SIRT1* encodes sirt1, a nicotinamide-adenine dinucleotide- (NAD^+^^−^) dependent HDAC, and deacetylates multiple substrates including transcription factors, histones, and enzymes^58^. This gene has been implicated it MDD in multiple recent studies. For example, Libert et al. and Lei et al. respectively, found that mice lacking *sirt1* in the brain exhibited depression-related behaviors^17,18^. In addition, Abe-Higuchi et al. found that chronic stress reduced the activity of sirt1 in the dentate gyrus (DG) of murine hippocampus, thereby contributing to the onset of depression-like behaviors^59^. When sirt1 activation was rescued in these mice, the depression-related phenotypes were significantly alleviated, while pharmacological inhibition of hippocampal sirt1 function resulted in increased depression-like behaviors^59^. Likewise, Lo Iacono et al. examined the *sirt1* mRNA expression in an adult “depressed” mice model established with juvenile isolation stress, and found significant reduction of *sirt1* expression in both the brain and peripheral blood mononuclear cells^60^. In the present study, we observed significant association of *SIRT1* risk allele with lower mRNA of this gene in human tissues, which was consistent with the diagnostic analysis which found decreased *SIRT1* expression in MDD patients. Our results are thus in line with the above studies in murine models, and reduced level/activity of sirt1 is therefore a potential risk factor for MDD. However, it should be noted that animal model studies have also obtained varied results. For instance, Ferland et al. reported that rats exposed to chronic stress had higher protein levels and activities of sirt1 in the hippocampal CA3 and DG regions^61^. Kim et al. observed increased sirt1 expression in the nucleus accumbens (NAc) of stressed mice^62^. While these data may seem inconsistent, possible explanations have been raised, including varied genetic backgrounds of studied animals and different stress exposure protocols^58^. Besides, previous studies also reported significant impact of circadian control machineries on sirt1 activity^59^, it is therefore speculated that discrepancies in the time of experiment conduction might have contributed to the varied results, especially that of the behavioral studies^58^.

Majority of the MDD studies involving *sirt1* focused on mPFC, hippocampus, and NAc, the brain regions well-known to facilitate emotion control and cognition. Using neuroimaging results obtained from human subjects, we also expanded the understanding of potential MDD mechanisms underlying the genetic risk conferred by *SIRT1* to an additional brain region. We show that *SIRT1* is likely involved in cerebellar structure and development, especially in the left posterior cerebellar lobe. While the precise function of *SIRT1* in this brain region is unclear, the significant association of *SIRT1* with GMV in the left posterior cerebellar lobe after multiple corrections in two independent samples is unlikely observed by chance. In agreement with this, sirt1 has been reported to act as an upstream regulator of Sonic hedgehog (SHH) pathway in normal and oncogenic neural development^63^, and SHH signaling plays a vital role in the cerebellar development^64^, providing hints for appropriate neurodevelopment in MDD. On the other hand, sirt1 is involved in mitochondrial biogenesis^65^, the process likely related to cerebellar development and MDD pathogenesis^66–68^. We thus hypothesize that rs12415800 may confer risk of MDD via reducing *SIRT1* expression and therefore abnormal cerebellar development. Indeed, evidence for the involvement of cerebellum in the neurobiology of MDD and cognition has emerged^8,45–47^. Compared with other brain areas, cerebellum has a longer developmental timeline, making it vulnerable to a series of internal and external risk factors^69,70^. Additionally, there are extensive connections between cerebellum and cerebral cortex^7,71^, the brain area consistently highlighted in recent genome-wide meta-analysis of depression^11^. It is thus reasonable to assume that cerebellar abnormalities may lead to deficits in cortical developmental^72,73^, and thereby contributing to depression. In summary, the gray matter reduction in the left posterior cerebellar lobe might affect the prefrontal–cerebellar circuit and results in the emotional and cognitive deficits in MDD. However, it is also possible that the association of rs12415800 with cerebellum may reflect a pleiotropic effect of *SIRT1* in complex traits and human health. For example, *SIRT1* expression has been reported to decrease in patients with autistic spectrum disorder (ASD) compared to healthy controls (https://cells.ucsc.edu/?ds=autism)^74^, and reduced cerebellar gray matter in the ASD patient has also been reported^75^. These data suggest that the association of rs12415800 with cerebellum may be shared in many psychiatric conditions.

We identified the associations between MDD risk allele and *SIRT1* mRNA expression in human brain and blood tissues, which is in agreement with the hypothesis that noncoding risk loci of complex diseases tend to affect gene expression in relevant tissues^32^. This hypothesis has been validated in various European samples. However, owing to the difficulties of brain tissue collection, genome-wide transcriptome analysis in Chinese brain samples has not been extensively published yet. Here, using candidate gene qRT-PCR analysis, we found that the MDD risk allele might contribute to *SIRT1* mRNA variation in Chinese human brains. Although our Chinese sample size is much smaller than those of published European studies, we believe that this brain sample still provide valuable information that promotes our understanding of the molecular mechanisms of MDD and other psychiatric disorders. However, the association of rs12415800 with *SIRT1* expression is not robust either in brain or blood tissues, it is thus possible that there exist additional variants in LD with rs12415800 showing stronger associations with *SIRT1* expression. This speculation is warranted especially considering that rs12415800 is located in the intergenic region, and functional predictions using HaploReg v4.1 suggested that it was unlikely a functional SNP^76^. Besides, although we identified the eQTL associations between rs12415800 and expression of *SIRT1* in the brain and blood tissues, we noticed that SNPs in high LD with rs12415800 also spanned additional genes, such as *CTNNA3*, *DNAJC12*, *HERC4*, and *MYPN*. Although the functions of those genes in MDD pathogenesis are less investigated compared with *SIRT1*, we cannot exclude the possibilities that those genes may also be relevant to MDD genetic risk and participate in its pathogenesis. For example, *CTNNA3* has been reported to preferably expressed in the cerebellum, although there were no overt cerebellar morphological changes in *CTNNA3* knockout mice compared with wild-type mice^77^. Further investigations of these genes are also necessary.

The non-significant association of the other COMVERGE MDD GWAS SNP rs35936514 in our *Han* Chinese sample is not unexpected. There are several explanations for this failure of replication. First, our sample was not as large as the CONVERGE sample, and a resultant lower statistical power of our MDD sample might cause this inconsistency. Moreover, there are several studies demonstrating the population stratification between different regional *Han* Chinese samples, and some genomic loci exhibit differential association statuses with diseases or traits between regional *Han* Chinese populations, which might affect the replication of results between different studies^78,79^. In fact, failures in replications of GWAS loci for psychiatric disorders in *Han* Chinese have already been reported several times^80,81^.

Notably, there are several limitations and we are cautious in the interpretation of the present results. First, we noticed that *p*-value of the association between rs12415800 and MDD in our sample did not achieve the genome-wide level of statistical significance (*p* = 5.00 × 10^–8^), which was likely caused by the limited sample size and the "winner’s curse" effect that the genetic effects of new association findings tend to be overestimated in the discovery study^82^. Second, despite the MDD samples we utilized have been reported previously and demonstrated to be effective in identifying the authentic genetic risk effects, it is acknowledged that the analysis of population stratification in this sample is lacking because the genome-wide SNP genotypes are unavailable at present. Although the cases and controls were randomly selected, further analysis after removing the effects of population substructure might further strengthen the conclusions. Third, whether the expression of *SIRT1* was also altered in the left posterior cerebellar lobe of MDD subjects, the brain region highlighted in our imaging analyses, remains unclear. Further analyses of gene expression analyses and functional studies involving with cerebellar tissues would strengthen the present study. Finally, although we observed strong statistical associations between cerebellum structure and MDD risk alleles, the mechanisms for this link remains opaque, and future investigations are needed to characterize the function of cerebellum in MDD.

In conclusion, we have confirmed a MDD risk gene *SIRT1* in *Han* Chinese population, and have identified a novel neural and molecular mechanism underlying genetic risk associations. In addition, we report the novel finding that individuals carrying MDD risk alleles show shifts in cerebellar structure even in healthy populations, and the cerebellum therefore might be relevant to the MDD risk linked to aberrant *SIRT1* expression. These results together provide new insights into the pathogenesis of MDD.

## Supplementary information

Supplementary Material
