# Supplementary material for: The depression GWAS risk allele predicts smaller cerebellar gray matter volume and reduced SIRT1 mRNA expression in Chinese population
Source: Transl Psychiatry. 2019 Dec 9;9:333. doi: 10.1038/s41398-019-0675-3 (PMC6901563; doi:10.1038/s41398-019-0675-3)

**Table S1. Genotypic distributions of rs12415800 and rs35936514 in Chinese MDD samples**

| **CHR** | **SNP** | **Position** | **Location** | **A1** | **A2** | **TEST** | **Case** | **Control** | **P-value** |
| --- | --- | --- | --- | --- | --- | --- | --- | --- | --- |
| 10 | rs12415800 | 69624180 | Intergenic | A | G | GENO | 354/946/524 | 582/1462/968 | 0.033 |
| 10 | rs35936514 | 126244970 | Intron | T | C | GENO | 136/665/996 | 224/1117/1639 | 0.947 |

**Note:**

CHR, chromosome; SNP, single nucleotide polymorphism; A1, allele 1; A2, allele 2.

**Figure S1. SNPs showing LD with rs12415800 in Chinese (CHB) and Europeans (CEU)**


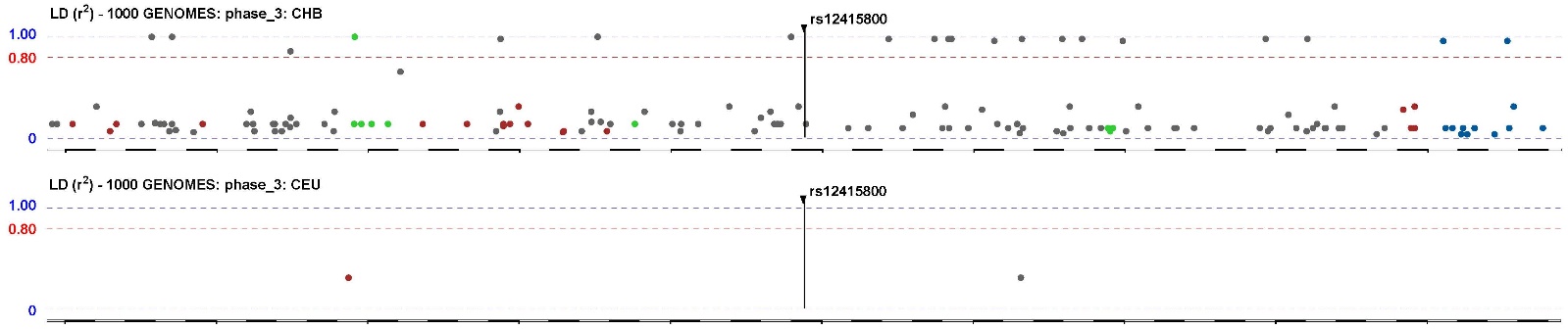

Supplement: Supplementary file 1 — Supplementary Material [file 41398_2019_675_MOESM1_ESM.docx]
